# Hopital la Salpetriére

**Published:** 1833-05

**Authors:** 


### HOPITAL LA SALPETRIÈRE.

*Case of Congenital Bulimia.* Communicated by Dr. Descuret.

Anne Denise L'Hermina, born at Noyou, 23d July, 1786, from the first moments of her life, was remarkable for her voracity, exhausting her nurses, and sucking more than four children of her own age. Towards her seventh year the menstrual flux began, and with this function were developed the other attributes of puberty. As she advanced to her tenth year her gluttony kept pace with her age, and obliged her twice to quit her foster-parent, because she ate the bread of all the children in the school.

Wandering then from village to village, she supported herself with raw greens and bread, which she received from charitable persons. On returning to Noyou for the third time, she set up a little school, and only required in payment bread, of which she consumed ten pounds a-day; but, quitting soon after a profession which did not relieve her appetite, she went to St. Quentin, to rejoin her elder sister, who placed her in the service of a gardener, who kept her on short commons; and afterwards she lived at an inn, where she found an ample supply.

Having by a blow hurt the right breast, she came to Paris for advice, but, before entering the hospital, she was twice arrested for stealing, at bakers', several loaves, which she devoured at the moment. Conducted to St. Louis' Hospital, she was affected for seven months with a bloody discharge from her wound, and, in spite of this hemorrhage, which the attention of the surgeons could not entirely stop, she was regular. A vomiting of blood, to which she was subject for some years, continued to take place periodically. She was afterwards transferred to the Venereal Hospital; and, on quitting this establishment, she offered her services to more than one person, who failed not to discharge her as soon as they perceived her bulimia, and the epileptic attacks to which she had been subject since seven years of age. Abandoned to her unfortunate lot, she wandered in Paris, living upon alms, and eating the refuse of aliments which she found at the doors. The assistance she received being insufficient for her appetite, she entered a brothel, from which she was taken by a charitable person, at whose recommendation many physicians tried, without avail, a quantity of remedies to restore her to health.

At this epoch, Denise was placed in the Salpetrière, in the epileptic division, where she was attended by Messrs. Esquirol and Amusat. Her habitual hunger was then satisfied with from eight to ten pounds of bread daily. Her sleep was very short; she hardly ever took liquids, except during the epileptic attacks. Her evacuations were rare, and occasionally bloody. She had vomiting of blood two or three times in a month. Her *great hunger* seized her nearly as often; she then eat, during the night, as much as twenty-four pounds of bread. At the commencement of the access she lost cognisance, and, as soon as she recovered it, she threw herself on her bread, and became so furious if they contradicted her in this imperious want, that she would bite her clothes, and even her hands, and returned only to reason after having entirely calmed her hunger. At this time the epigastrium was the seat of pain, which pressure augmented; she also felt a body rise in the œsophagus, which she compared to a large leaf of a tree. She seemed to be forcibly bound about the breasts; she became bathed in a cold sweat; then, on making efforts to reject the body which oppressed her, the leaf descended into her stomach, remounted soon more or less high; at last vomitings of clotted black blood, floating in blood more clear and unmixed with aliments, relieved her, and the appetite took its habitual type, until the same occurrences came to manifest anew themselves.

These attacks brought her frequently to the infirmary, where M. Rostan made her follow several kinds of antiphlogistic treatment. Some months after, she went out of the Salpetrière, and experienced the same crises frequently, until the month of February 1823, at which time she came to consult me. She complained of an insupportable itching at the nose, navel, and arms; she had the pupil very dilated; pulse regular, without fever; skin cool, tongue loaded, and mouth bitter. I asked her if ever she had passed worms, to which she answered in the negative. She then took two ounces of castor-oil, with an ounce of syrup of lemon; and the following day brought to me several fragments of tænia she had passed, and said the unpleasant symptoms had ceased.

From this time the hunger of Denise decreased sensibly; she consumed but five pounds of bread, and two or three strong soups, each day. The *great hunger* she had experienced periodically on the 9th February for five years did not occur this time, nor did it again take place till 1828.

Denise had then, to my knowledge, three sorts of hunger: 1, the hunger which, from 1820 to 1822, was appeased by twelve pounds of aliments in twenty-four hours; 2, that hunger which took place three or four times a-month, more frequently still on the least contradiction, and during which she ate from twenty to twenty-four pounds of bread; 3, *her great hunger*, which occurred on the 9th February, for five years in succession, and once on Good Friday, because she had thought of fasting. It was then she devoured, in twenty-four hours, from thirty to thirty-two pounds of aliments, as much bread as soup, eating and vomiting blood alternately, until she was completely exhausted. One year, being in the kitchen of the Marchioness de Latour de Pui, on the 9th February, Denise was seized with her *great hunger*, and swallowed up in a few minutes the soup destined for twenty guests, and twelve pounds of bread.

On being taken home, she continued eating during a part of the night, and almost all the next day.

As before mentioned, from February 1823, the appetite of Denise diminished considerably, which should in part be attributed to the expulsion of the tenia: I say, in part, for from this moment she took to drinking alcoholic liquors in frightful quantities; she drank every two hours either a glass of wine or of brandy, pretending that liquids nourished her more than solids. It may easily be conceived how numerous were the ill consequences produced by such irregularities of regimen. The worst of all was the suppression of the menses; this occurred in 1826, and it was often necessary to bleed locally and generally, though these means gave only momentary relief. On the other hand, Denise, whose stomach was continually over-exerted by stimulating drinks, began to have singular tastes. Thus, occasionally she ate raw cat's-meat, and cleaned her teeth by browsing on the grass, which ordinarily she digested pretty well. On the 1st July, 1828, she went to her favourite pasture, gathered a basket of herbs and buttercups (Ranunculus acris,) which she ate for supper. During the night she was taken with violent cholic, which she in vain tried to assuage with hot wine and brandy.

The following days the pains diminished so that she could go out, but was obliged to take to her bed soon after, and I was sent for on the 12th, in the morning.

I found her affected with jaundice, for which she was treated, and observed my directions for a few days; but, on one occasion, she gorged herself with wine and brandy. On the 5th August, she drank a bottle of brandy, and died within twenty-four hours after.

*Autopsy.* The stomach was small; its mucous membrane presented in parts, as well as that of the intestines, some inflamed points. We found no kind of worms. The liver was very voluminous; the bladder and uterus very small; Denise had not had any children. The contents of the chest healthy. The head was not opened, as it was to be preserved. It presented a remarkable peculiarity; the condyles of the inferior maxilla were almost entirely destroyed, which may easily be accounted for by recollecting that mastication had been continuous for nearly forty-two years. Denise was of ordinary height and size, of a purely sanguine constitution, though her limbs were of a pale whiteness and softness, which indicated the excess of the cellular tissue over the strength of muscles. Her gait, voice, and gestures, partook more of the man than of the female. Her eyes, though of a clear blue, had the character of those of the hyæna. The sexual feeling was for a long time excessive, and she experienced a slight attack of epilepsy whenever she had connexion.

Her conversation was brusque, unconnected, turning mostly on her appetite, and was but a tissue of lies. On one occasion she asserted she had eaten seventy-two pounds of bread in twenty-four hours, whilst, on the most exact calculation, it was found that thirty-six pounds, including soup, was the utmost she had ever taken. She acknowledged to have been in the habit of drinking every morning a glass of wormwood cordial, when she gorged herself constantly with strong liquors. Flowers had for her an irresistible attraction; many times has she followed for entire hours persons who have carried them.

Active, obliging, charitable, Denise gave at times money to the poor, but never any bread. Often commissioned by individuals to receive considerable sums of money, and at the same time to make purchases, Denise showed constantly the most scrupulous trustiness on these occasions. Her probity was never shaken at the sight of gold; but it failed her before a morsel of bread. One morning, in the streets, she observed a mason engaged, who had deposited his bread upon a stone near him. Denise had both money and bread in her basket; nevertheless, she ran away with the bread of this man as fast as she could. Some days after she came to me, and related the circumstance, asking me if she ought not to send five francs to the mason, whose dwelling she was acquainted with. I approved of her intention, and proposed that she should join a loaf with the money, to replace that she had taken. At the word, her features altered, swelled, the lower lip trembled with rage, her eye sparkled, foam ran from her mouth: "I will send him ten francs," said she in an angry tone, "fifteen, if you will, but never shall he have of me a morsel of bread."

Easily excited at all times, she became much more so after she had given herself up to drink: she changed her lodging beause a cat had, from the roof, looked at a soup she had put in the window to cool. On another occasion, her soup being partly upset in the fire, in order not to lose what remained, she swallowed it boiling, which occasioned that day five vomitings of blood. Finding herself one day shut in with a Mademoiselle D., in the library of the church of St. Geneviève, her first care was to examine the contents of the basket she was in the habit of carrying constantly, and finding in it only a pound of bread, fear of want carried her away so that she spoke in a most strange way, not knowing, said she, to what extremity hunger might lead her.

Already had she attempted to climb the walls to get to a high window, when, to her great joy, some one came to open the door. On another occasion, at my own house, I was bleeding a patient, when a large piece of bread which she held under her arm fell into the bason which received the blood; she took it out in great haste, and devoured it all covered with blood.

To resume, we might assert that this woman had lived entirely for digestion. During the first months of her life, she exhausted several nurses; as a child, she devoured the bread of her school-fellows; as an adult, she ate day and night; becoming less voracious, she was continually in a state of drunkenness; struck with death, she wished to recover only to eat; at last, some moments before death, being no longer able to eat bread, "because," said she,

"*le pain produisait mal au cœur,*" she forced her sister to eat near her, almost in her mouth, and died, saying "Since the good God wishes I should no longer eat, at least I may have the pleasure of seeing eating."

Considered in a phrenological point of view, the cranium, which I have preserved, presented the organs in the following order of predominance: 1. Alimentiveness;* 2. Amativeness; 3. Combativeness; 4. Secretiveness; 5. Philoprogenitiveness; 6. Constructiveness; 7. Attachment; 8. Conscientiousness; 9. Firmness; 10. Individuality; 11. Form; 12. Locality; 13. Circumspection; this last very little developed.

Compared with fifteen crania, taken by chance by M. Broussais and myself, but two offered the organ of alimentativeness developed as large as one-third less than this organ in Denise, and these were the crania of men.—[*Broussais, Ann. de Medicine.*]

* The part of the brain in which this organ is situated is the most antero-lateral convolution of the middle lobe, immediately posterior to the fissura magna sylvii, at its lower part; when large, it swells out the squamous portion of the temporal bone above and in front of the external auditory meatus, and, with a little attention, may be distinguished under the temporal muscle, when its fibres are not in action; allowance being made for the obvious difference of thickness of the muscular fibres of the integuments covering them.

